# Advantages and disadvantages of self-determination support strategies for people with severe or profound intellectual and multiple disabilities: A Delphi study

**DOI:** 10.1371/journal.pone.0349403

**Published:** 2026-05-19

**Authors:** Pála Björk Kúld, Noud Frielink, Carlo Schuengel, Petri Embregts

**Affiliations:** 1 Department of Tranzo, Tilburg School of Social and Behavioral Sciences, Tilburg University, The Netherlands; 2 Department of Clinical Child and Family Studies, Vrije Universiteit, Amsterdam, The Netherlands; University of Tartu, ESTONIA

## Abstract

**Background:**

The specific advantages and disadvantages of strategies that caregivers use to support self-determination in individuals with profound intellectual and multiple disabilities need to be known. This study explored consensus on advantages and disadvantages of support strategies from the perspective of users.

**Method:**

A four-round Delphi study was conducted with 10 relatives and 13 professionals experienced in self-determination support. In Round 1, qualitative data were collected and thematically analysed. Subsequent rounds used Likert scales and participant feedback to explore consensus.

**Results:**

Thematic analysis of Round 1 produced 79 themes, on which 72 consensus was reached across later rounds. On 63 themes, consensus was found across both groups. Users reported advantages such as mutual trust, reciprocal learning, and a more unified and efficient approaches to support self-determination. For individuals with disabilities, advantages included improved health, participation, and increased influence over their lives. However, disadvantages included preconditions that are often unmet in practice, such as consistent use of communication aids, sufficient time, and stable staffing.

**Conclusion:**

Promoting self-determination in this population requires attention to their interactions with support networks and the quality of collaboration. Each support strategy has specific advantages and disadvantages that should be examined in light of the diversity within this population and their networks. Future research and practice could address identified barriers and build on advantages of support strategies by evaluating their applicability and relevance in individual cases.

## Introduction

Every day, family members and healthcare professionals who support people with severe or profound intellectual and multiple disabilities face the critical challenge of fostering self-determination [1]. For this population, self-determination is associated with reduced self-injurious behaviours, increased adaptive functioning, community participation, and overall quality of life [2–5]. Historically, self-determination was assumed to require high cognitive capacities [6]. However, in the last two decades, the potential for self-determination among individuals with severe or profound intellectual and multiple disabilities, contingent on adapted environments, has gained increasing recognition [7]. This shift aligns with the social model of disability and the United Nations Convention on the Rights of Persons with Disabilities (UNCRPD) framework, emphasizing autonomy, dignity, and free will as fundamental human rights [8,9].

Despite these advances, the assumption that this population experiences lower levels of autonomy and participation compared to other populations prevails [10,11]. People with severe or profound intellectual and multiple disabilities usually have severe or profound intellectual impairments (IQ < 20–35), and complex neuromotor and sensory disabilities [12]. Their communication is often described as pre- or protosymbolic, expressed through subtle body movements, muscle tension, vocalizations, and idiosyncratic behaviours with individual and across variations. These variations may also contribute to the variations in communication, language and motor capabilities seen across people within this group [12–14].

Individuals with severe or profound intellectual and multiple disabilities are typically highly dependent on others for daily care and support and often have limited verbal communication. As a result, their preferences and intentions are frequently interpreted and mediated by significant others. Most individuals with severe or profound intellectual and multiple disabilities grow up within their family environment, where self-determination is often intuitively supported by family members [1]. Parents gradually develop expertise in understanding their child’s behaviour, needs, and preferences and frequently translate this knowledge to others involved in care [15]. Siblings are also commonly involved in caregiving and may eventually assume parents’ roles over time [16]. Throughout the life course, family members typically receive assistance and guidance from healthcare professionals, who fulfil various roles in supporting the individual [17]. Consequently, the degree, frequency, and nature of family members’ and healthcare professionals’ involvement and collaboration may change over time [16].

The communication forms used by people in this target group, while meaningful, are often difficult to interpret accurately, which can hinder the identification of opportunities to support self-determination [9,18]. Nevertheless, emerging evidence suggest that changes to the social environment may support self-determination. Individuals with severe or profound intellectual and multiple disabilities may experience self-determination when supporters, both family members and healthcare professionals act as facilitators rather than gatekeepers of autonomy [7]. Central to this approach are person-centered strategies, grounded in the deep, often tacit, knowledge that supporters have of the individual [7]. Such relationships are key to recognizing preferences, interpreting subtle cues, and fostering participation in decision-making [18–19]. Other reported practices include promoting choice-making, facilitating communication, providing assistive technology, and cultivating sustainable support networks [20]. Finally, collaborative approaches of family members and professionals are assumed to shape environments supportive of self-determination [21]. Furthermore, as the present study was conducted in the Netherlands, it is relevant to consider the national care context in which it is embedded. Dutch disability care policies and programmes increasingly emphasise the promotion of self-determination, including for individuals who are highly dependent on others [21,22]. This socio-cultural and policy context may shape both professional practices and family expectations regarding autonomy and support. At the same time, cultural differences and variations in service organisation across countries should be considered when interpreting the findings, as their transferability to other national contexts may be limited [23].

Given the range of potentially effective strategies, it is important to consider their relative advantages, guided by established theoretical frameworks. Self-determination among people with disabilities may be understood by integrating insights from Self-Determination Theory (SDT) and Causal Agency Theory (CAT). SDT and its mini-theory of basic psychological needs posit that the pursuit of autonomy, competence, and relatedness constitutes self-determination, which is subjectively experienced in the form of autonomous motivation and wellbeing [24]. CAT offers a framework for understanding how people become self-determined, in the sense that they develop actions and beliefs that allow them to take charge of their own lives. Previous research suggests that the needs identified by SDT can also be used to understand self-determination in individuals with severe or profound intellectual and multiple disabilities [18]. However, operationalising these needs within this population is challenging, as internal experiences are often not directly accessible and must be interpreted through proxies who rely on non-symbolic behaviours [26-27]. CAT complements SDT by emphasising that self-determination is not merely about exerting control, but also about learning how one’s actions bring about desired changes [27]. From this perspective, the function of an action for the individual, specifically, whether it enables them to influence their environment is central. Thus, while SDT focuses primarily on the fulfilment of psychological needs, CAT highlights the development of observable behavioural and relational processes through which these needs may be expressed and supported. For individuals with severe or profound intellectual and multiple disabilities, whose inner experiences are often difficult to assess directly, this integrated perspective is particularly relevant. Self-determination should therefore be examined within individuals’ interactions with their support networks, taking into account both psychological needs and the contextual conditions that enable causal agency.

However, although previous studies have identified several strategies that may support self-determination in this population, important questions remain unanswered. Existing research has helped to describe relevant strategies and to relate them to broader theoretical frameworks [25,26]. However, a key step is to discern the relative advantages and disadvantages of these strategies across individuals, situations, and support contexts. In addition to these theoretical and methodological gaps, support strategies may also be constrained in practice. The contexts in which self-determination can be supported are highly diverse, and strategies that appear useful in one situation may be less feasible or effective in another [28–32]. Their applicability may depend on individual factors, such as health status or communication preferences [21,31], as well as contextual factors including caregiver workload, organisational rules, and institutional routines [7,28]. Yet, the literature offers only limited insight into how relatives and healthcare professionals experience such constraints in daily practice, and how these may shape the perceived value of different strategies.

To conclude, important open questions include which support strategies are most beneficial, what barriers may hinder their use, and how judgements on these questions may differ between stakeholder groups. Because people with severe or profound intellectual and multiple disabilities are often unable to express their inner experiences directly [7], insights from relatives and professionals who apply these strategies are essential [10,21]. This study therefore focuses on how these stakeholders perceive the advantages and disadvantages of current support strategies. Prior research suggests that relatives and professionals may differ in how they support self-determination, drawing on either intuitive, relational knowledge or formal training and policy [1]. Examining both perspectives may help clarify what works for whom, under what conditions, and with what possible limitations. By synthesising experiential and practice-based knowledge, this study aims to answer the research question: What are the advantages and disadvantages of support strategies used to promote self-determination in people with severe or profound intellectual and multiple disabilities?

## Method

### Study design

The current study employed the classic Delphi design [33], known for its potential to find expert consensus on understudied topics with divergent opinions [34,35]. This method involves multiple iterative rounds of anonymous feedback among participants, minimizing the influence of hierarchy and groupthink, thereby promoting equitable collaboration. This collaborative process can lead to detailed and comprehensive knowledge about topics under investigation. The Delphi method has been effectively applied in various fields, including intellectual disability research [25,36–38]. Additionally, an Advisory Commission of experts by experience recommended interviews in the first round instead of a questionnaire, to avoid burdening participants with writing requirements on an underexplored topic more suitable for an open discussion.

Ethical approval was obtained from the Ethical review board of Tilburg University [TSB_RP1186].

### Participant recruitment

A purposive expert sampling method was employed (Powell, 2003). In Delphi studies, a panel of 10–15 experts with a 70% response rate per round is typically sufficient to yield reliable results [39]. Taking into account an expected attrition rate of 20–24 percent [40], the recruitment target was set at 24 participants.

Participants were recruited through the contact persons of The Academic Collaborative Center Living with an Intellectual Disability (AWVB). These contact persons from affiliated healthcare organisations approached eligible individuals based on predefined inclusion and exclusion criteria. These criteria required participants to be relatives or healthcare professionals (16 years or older) involved in the care of individuals with severe or profound intellectual and multiple disabilities, as defined by Nakken and Vlaskamp [41]. This definition includes a combination of intellectual disabilities (IQ below 20–35), neuromotor and sensory impairments, and a developmental age below five years [42]. A minimum calendar age of three years was set for the person with disabilities, reflecting the typical developmental onset of autonomy between 18 months and three years, and the beginning of conscious parental autonomy support [43]. Relatives included first- or second-degree family members, friends, or legal representatives. Healthcare professionals were required to have at least two years of experience working with the target population [1]. A heterogeneous sample was sought to improve the generalisability of the findings [39]; thus, no restrictions were placed on the occupational roles of healthcare professionals. To maximise the likelihood of relevant expertise, participants were purposively selected based on their direct experience in supporting individuals with severe or profound intellectual and multiple disabilities. Inclusion criteria were defined according to the participant’s relationship to the support recipient: either being a first- or second-degree relative or having at least two years of professional experience working with the target population. In addition, participants received a brief overview of the identified support strategies and confirmed their familiarity with these practices. They were also provided with a short-written introduction to self-determination as conceptualised within Self-Determination Theory (SDT) to ensure a shared conceptual basis. Although no formalised or standardised criteria for expertise were applied, consistent with previous Delphi research [33], sustained practical involvement in supporting self-determination was considered sufficient to qualify participants as expert informants for the purposes of this study.

Interested participants received an email containing an information letter explaining the study’s purpose and procedures, and an informed consent form. The form clarified that participants could withdraw their participation at any time without consequences or needing to provide a reason. Participants signed the consent form, filled in a brief demographic questionnaire and returned these through e-mail. Recruitment of participants took place from May 1^st^ to October 8th, 2024, when participants had returned their signed consent forms, interviews with those participants could already take place, therefore the timing of recruitment and data collection collided. Interviews took place from 6^th^ of June to 17^th^ October 2024 and questionnaire data were collected for all rounds between 19^th^ of November 2024–16^th^ of April 2025. During and after data collection, all data containing privacy sensitive information were pseudonymized. Data can only be traced back to the person by the means of a key file located on a protected drive of the University, only accessible to the executive researchers. All participants were compensated with a 25-euro gift card after completing participation.

A total of 23 participants enrolled in the study; 10 relatives and 13 healthcare professionals. As all recruitment avenues where exhausted at this point, recruitment was concluded. Relatives included five mothers, three fathers, one brother, one sister, and one friend/legal representative. Their ages ranged from 25 to 75 years (mean = 58, SD = 14.7). Their family members with disabilities included seven males and three females, aged 9–68 years (mean = 26, SD = 16,7), all with severe to profound intellectual and multiple disabilities. Healthcare professionals included 12 females and one male, ranging in age from 22 to 59 years (mean = 41, SD = 11.8). Their work experience with the target population ranged from 2 to 39 years (mean = 14.7, SD = 11.4), and they occupied a variety of professional roles (see Table 1 for further details).

**Table 1:** Participants demographics.

Family members	
Mother & father together	1
Mothers	5
Fathers	3
Sibling	2
Friend/legal representative	1
Gender	
*Male*	5
*Female*	6
Age range (years)	25-75
**Healthcare professionals**	
Direct support staff	4
Team-leaders	2
Project managers	1
Nurse practitioners	2
Care coordinator	1
Project quality manager	1
Treatment coordinator	1
Therapist	1
Gender	
*Male*	1
*Female*	12
Age range (years)	22-59
Work experience (years)	2-39

### Delphi procedure

#### Preparation phase.

Prior to initiating the four-round Delphi process, an overview of strategies used to support self-determination in individuals with severe or profound intellectual and multiple disabilities was compiled, similar to approaches in previous Delphi studies [36,37]. This overview was based on four key sources: 1) a systematic review of intervention studies [20], 2) a concept mapping study [21], 3) an earlier Delphi study on self-determination for this population [25], and 4) Dutch healthcare practice literature [44,45]. The overview prioritized empirically supported interventions, with a particular focus on those used in the Netherlands, while also integrating international literature.

The first author screened all materials and extracted interventions specifically targeting self-determination, using terminology consistent with prior work [20,21,25]. A comparative matrix was created to identify overlapping strategies, particularly within core domains such as autonomy and choice-making. To validate this overview, an online advisory group meeting was held with six specialists (three relatives, two professionals, one with dual roles) who did not participate in the final Delphi study. Prior to the meeting, they received an outline of the proposed strategies and the Delphi procedure. Feedback focused on the applicability, clarity, and relevance of each strategy. Revisions were made from their input. The matrix is available in the supplementary materials.

This process resulted in the identification of six core strategies: 1) Equal cooperation of relatives and healthcare professionals to create an environment optimal for self-determination; 2) Build and maintain sensitivity; 3) Purposeful application of total communication; 4) Support and encourage choice making; 5) Implement technology and aids; and 6) Create and maintain a network of acquaintances around the person. A summary is provided in Table 2; further details are available upon request.

**Table 2:** Overview of self-determination support strategies presented to participants in round 1.

Strategy	Description
1.Equal cooperation of relatives and healthcare professionals to create an environment optimal for self-determination.	Collaborative efforts to understand the individual’s behavior, communication, preferences, and needs. Diverse expertise of all parties is equally valued, fostering an environment promoting self-determination.
2.Build and maintain sensitivity	Observe and interpret subtle behaviour (e.g., vocal sounds, body movements, muscle tension) to accurately recognize and respond.
3.Purposeful application of total communication	Utilize communication methods/assistive technologies tailored to individuals communication style, developmental level, abilities, and context. Regular re-evaluations to accommodate evolving needs.
4.Support and encourage choice making	Identify individuals’ ability to make choices. Align choice-making opportunities with developmental level, communication- and sensory abilities, and specific contexts.
5.Implement technology and aids	Assess/apply suitable assistive technologies (e.g., microswitches, optical pointers, tablets, AAC systems) to facilitate communication, choice-making, and monitoring of physical/mental well-being.
6.Create and maintain a network of acquaintances around the person	Establish/sustain a network of acquaintances familiar with the individual to share and transfer knowledge.

*Notes; AAC systems = augmentative and alternative communication systems.*

#### Delphi rounds.

To explore the perspectives of relatives and healthcare professionals on the advantages and disadvantages of six strategies to support self-determination in individuals with severe or profound intellectual and multiple disabilities, a four-round Delphi design was employed. Based on advice from the advisory group, the first round consisted of individual online interviews in Microsoft Teams. The interviews lasted around 30–45 minutes where participants explained their experienced advantages and disadvantages of each strategy consecutively, with flexibility to revisit strategies if needed. The subsequent three rounds were conducted through online questionnaires distributed via email. Questionnaire completion times ranged from approximately 10–30 minutes. In line with previous research [1], intervals of five weeks were maintained between rounds to allow for analysis and preparation. Each round was planned to remain open for 14 days, with reminder emails sent after one and two weeks. Due to delays in retrieving the filled in questionnaires, interval between rounds was extended. The procedures and focus of each round are described in detail below.

**Round 1: Item generation through interviews.** The objective of the first round was to generate a broad set of perceived advantages and disadvantages for each of the six strategies. Each interview lasted approximately 30–45 minutes and was guided by open-ended questions designed to elicit participants’ views and experiences related to the proposed strategies. Prior to the interviews, participants reviewed the strategies in detail. Interviews were audio-recorded with consent (verbal and written) and transcribed verbatim. The interviews yielded 619 individual responses, which were thematically analysed and organized into 79 overarching themes (see Data Analysis section for further details). These themes encompassed both positive and critical perspectives and were subsequently carried forward to the second round.

**Round 2: Initial evaluation and consensus testing.** The second round’s objective was to evaluate the level of agreement with themes generated in Round 1. Participants received an online questionnaire via the Qualtrics platform, in which they rated each theme using a 5-point Likert scale ranging from 1 (strongly disagree) to 5 (strongly agree). Themes with at least 70% agreement or disagreement within each group were considered to have reached consensus and excluded from further rounds [46].

**Round 3: Re-evaluation with feedback.** The purpose of the third round was to further evaluate the remaining themes that did not reach consensus in Round 2. The questionnaire followed the same format as in the previous round, themes were not reformulated but included controlled feedback to facilitate reconsideration [35,36]. This feedback included each themes’ mode score from the group’s responses in Round 2. If a participant’s rating differed from the group mode, they were prompted to briefly explain their reasoning. This process aimed to promote deeper reflection and increased awareness of differing viewpoints within the panel. In this round, one relative could not participate due to personal circumstances, but returned for round 4. Additional attrition included three professionals (see Table 3. for further details).

**Table 3:** Participants response- and retention rate (%) in each round.

Participants	Round 1	Round 2	Round 3	Round 4
Relatives	10	10 (100%)	9 (90%)	10 (100%)
Professionals	13	10 (76.9%)	11 (84.6%)	11 (84.6%)

**Round 4: Final evaluation and consensus determination.** Round four served as the final opportunity for finding consensus. Themes that still lacked consensus after Round 3 were re-presented, this time with additional feedback. Participants were shown their own previous responses, the group mode, and a summary of anonymized qualitative arguments provided by participants whose ratings differed from the majority. This inclusion of peer reasoning provided further context for participants to re-evaluate their positions. After completing their ratings, themes that continued to fall short of the 70% agreement threshold were considered non-consensus items and excluded from the final results.

### Data analysis

#### Round 1 item generation.

In line with prior research [25,47], data from Round 1 were analysed using Braun and Clarke’s [48] six-step reflexive thematic analysis approach, allowing for a systematic identification of patterns in the qualitative interview data. First, the first two authors and an independent researcher familiarised themselves with the data by repeatedly reading all interview transcripts to develop an in-depth understanding of participants’ perspectives. Second, the first author and the independent researcher used ATLAS.ti software [49,50] to independently conduct open, inductive coding of each interview, focusing specifically on statements related to the perceived advantages and disadvantages of the identified support strategies. Codes were developed close to participants’ original wording and reflected meaningful features relevant to the research question. After coding all interviews, codes were compared and discussed with the third author. In line with a reflexive thematic analysis approach, these discussions were not intended to establish coding consensus as a marker of reliability, but to deepen interpretative engagement with the data and critically reflect on similarities and differences in coding. Reflexivity was considered throughout the analysis. The first author, who conducted the interviews, was familiar with participants’ non-verbal expressions, vocal tone, and interactional context. While this provided valuable contextual sensitivity, it may also have shaped the interpretation of the data. To maintain a shared analytical basis between coders, the analysis focused primarily on the transcript content available to both coders, while recognising that interpretation remained shaped by the researchers’ perspectives and involvement in the study.

Third, related codes were grouped into preliminary themes by the first and second authors and the independent researcher. While some codes clustered into coherent themes, others were initially categorised as miscellaneous and were further examined during the refining process. Fourth, the preliminary themes were reviewed against both the coded extracts and the full dataset to ensure coherence and alignment with the data. To enhance clarity and credibility, a peer debriefing procedure was conducted with two independent researchers. These researchers were not involved in the data collection or coding process. Both had extensive experience with qualitative research (especially thematic analysis) and professional and research expertise regarding the target group and the topic (self-determination), which enabled them to assess both the analytical coherence and the practical relevance of the theme labels. Their position outside the primary analysis process allowed them to provide critical reflection on the clarity, coherence, and internal consistency of the themes. Without access to the original codes or transcripts, they reviewed the theme labels, after which minor revisions were made based on their feedback. Finally, all authors contributed to defining and naming the themes to ensure conceptual clarity and accessibility for participants in the subsequent Delphi rounds. The finalised 79 themes were then incorporated into the Round 2 questionnaire analysed using the SPSS software version 29 [51]. All finalized themes were translated into English by a certified language center and are available in Tables 4–9.

**Table 4:** Agreement per group and rounds on advantages/disadvantages of strategy 1: Equal cooperation of relatives and healthcare professionals to create an environment optimal for self-determination.

Round	2		3		4	
**Advantages**	**Relatives**	**Professionals**	**Relatives**	**Professionals**	**Relatives**	**Professionals**
This strategy enables stakeholders to learn from each other’s knowledge and experience	100% agree	100% agree				
This strategy helps improve the quality of life of individuals with disabilities by providing more clarity, calmness and better support of self-determination	90% agree	100% agree				
This strategy makes relatives feel that healthcare professionals involve them, listen to them, and offer them advice and support in difficult situations	100% agree	100% agree				
This strategy helps relatives prepare for the transition when healthcare professionals take over the care duties for their loved ones	70% agree	50% neutral 50% agree		60% agree 40% neutral		90% agree
This strategy creates trust and connection between all involved, benefitting the quality of care that an individual receives	100% agree	90% agree				
**Disadvantages**						
This strategy may reveal differences in opinions and perspectives of relatives and healthcare professionals that can hinder their collaboration	40% disagree 30% neutral 30% agree	80% agree	77% disagree 11% completely disagree			
This strategy is more time-consuming in terms of reaching consensus	30% disagree 10% neutral 60% agree	30% disagree 30% neutral 40% agree	67% agree 33% disagree	70% agree	78% agree	
This strategy can lead to disagreement or uncertainty about task divisions	50% disagree 20% neutral 30% agree	20% disagree 30% neutral 50% agree	78% disagree	60% agree 40% neutral		70% agree
This strategy is sensitive to changes in personnel	70% agree	70% agree				
This strategy is difficult to implement if the relatives are not very involved	70% agree	80% agree				
In this strategy, an individual’s capabilities could end up not being taken into consideration sufficiently	50% disagree 20% neutral 30% agree	30% disagree 40% neutral 30% agree	78% disagree	50% neutral 30% agree 20% disagree		80% neutral 20% disagree

**Table 5:** Agreement per group and rounds on advantages/disadvantages of strategy 2: Build and maintain sensitivity.

Round	2		3		4	
**Sentence**	**Relatives**	**Professionals**		**Relatives**	**Professionals**	**Relatives**		**Professionals**
This strategy allows individuals with disabilities to express themselves better and helps the communication partner to respond to an individual’s needs and wishes	100% agree	100% agree						
When the knowledge that this strategy yields is shared within the team, the team adopts a more unified approach to interpreting behaviour and responding to it	90% agree	90% agree						
Implementation of this strategy by healthcare professionals reduces pressure on relatives	80% agree	10% disagree 40% neutral 50% agree			90% agree			
This strategy promotes interaction, job satisfaction and personal development among healthcare professionals	80% agree	100% agree						
This strategy strengthens the relationship of healthcare professionals with individuals and their relatives	80% agree	80% agree						
**Disadvantages**								
This strategy is very time-consuming and work-intensive for healthcare professionals	10% disagree 30% neutral 60% agree	40% disagree 20% neutral 40% agree		89% agree	50% disagree 10% complete disagree 20% agree 20% neutral			70% disagree
This strategy can be challenging to implement, as sensitivity is a skill that requires constant attention and refinement	70% agree	10% disagree 30% neutral 60% agree			70% agree			
This strategy can be difficult to implement because interpretations of behaviour can differ, depending on the person and the situation	70% agree	80% agree						
This strategy is difficult to implement due to workload, time constraints and staff shortages	30% disagree 20% neutral 50% agree	10% disagree 30% neutral 60% agree		56% agree 45% disagree	70% agree	90% agree		
This strategy is difficult to implement if one doesn’t know the individual well	70% agree	20% disagree 30% neutral 50% agree			70% agree			
In this strategy, having a large team size could yield many different opinions and interpretations of an individual’s behaviour	10% disagree 60% neutral 30% agree	20% disagree 30% neutral 50% agree		89% neutral	100% agree	89% neutral		

**Table 6:** Agreement per group and rounds on advantages/disadvantages of strategy 3: Purposeful application of total communication.

Round	2		3		4	
**Advantages**	**Relatives**	**Professionals**	**Relatives**	**Professionals**	**Relatives**	**Professionals**
This strategy gives individuals with disabilities structure and predictability	100% agree	70% agree				
This strategy allows individuals to express themselves to others more clearly and gain greater influence over their own lives	100% agree	70% agree				
This strategy is a low-key approach that helps reveal an individual’s preferences	90% agree	40% neutral 60% agree		100% agree		
This strategy strengthens the bond between individual and caregiver, lowering frustration and stress and potentially preventing self-harming behaviour	100% agree	80% agree				
This strategy promotes connection and contact, allowing healthcare professionals to better understand an individual and respond to their needs	100% agree	90% agree				
This strategy increases an individual’s self-esteem and autonomy	100% agree	80% agree				
This strategy helps determine what an individual needs to communicate based on his/her developmental level and capabilities	100% agree	90% agree				
This strategy makes individuals less dependent on others	60% agree	20% disagree 40% neutral 30% agree	89% agree	70% neutral 20% disagree 10% agree		70% neutral 20% disagree 10% agree
**Disadvantages**						
This strategy can cause an individual to experience confusion when healthcare professionals from different locations (e.g., daycare centres versus long-term care facilities) do not use communication aids uniformly	90% agree	80% agree				
This strategy can lead to under- or overestimation of an individual’s capabilities if communication aids are used that do not fit well with the individual’s developmental level	80% agree	90% agree				
In this strategy, deploying communication tools and learning how to work with them is very time-consuming	10% disagree 30% neutral 60% agree	70% agree	89% agree			
In this strategy, a successful implementation of aids is influenced by an individual’s health status and the motivation of all those involved	70% agree	90% agree				
This strategy can make it difficult for an individual to regain skills if the use of communication aids is temporarily interrupted (e.g., due to illness or injury)	50% neutral 50% agree	80% agree	89% neutral		89% neutral	
The disadvantage of this strategy is the potentially high cost of the communication aids and the required training programs and courses, which are also difficult to organize	30% disagree 20% neutral 50% agree	100% agree	89% agree			

**Table 7:** Agreement per group and rounds on advantages/disadvantages of strategy 4: Support and encourage choice making.

Round	2		3		4	
**Advantages**	**Relatives**	**Professionals**	**Relatives**	**Professionals**	**Relatives**	**Professionals**
This strategy stimulates the growth and autonomy of individuals with disabilities	80% agree	100% agree				
This strategy gives individuals more influence over their own life	90% agree	90% agree				
This strategy contributes positively to individuals’ mental and physical health	100% agree	90% agree				
This strategy promotes a client-centred approach, which improves the quality of care	100% agree	100% agree				
This strategy promotes job satisfaction and alertness among healthcare professionals	90% agree	100% agree				
This strategy promotes individuals’ social inclusion and societal involvement	70% agree	70% agree				
This strategy boosts individuals’ self-esteem and self-confidence	90% agree	80% agree				
**Disadvantages**						
Implementing this strategy is difficult due to time constraints and staff shortages	10% disagree 40% neutral 50% agree	20% disagree 40% neutral 40 agree	89% agree	70% neutral 20% disagree 10% agree		80% neutral
Implementing this strategy may limit an individual’s autonomy if his/her choices are misunderstood	10% disagree 40% neutral 60% agree	50% neutral 50% agree	100% agree	60% agree 40% neutral		90% agree
This strategy may not fit every level of the target group, which could limit its applicability (as not everyone is able to make choices)	80% agree	90% agree				
This strategy can demand a lot of energy from an individual, increasing the risk of overestimating his/her capabilities	20% disagree 40% neutral 40% agree	90% agree	78% neutral		78% neutral	
This strategy may make things difficult for healthcare professionals if an individual’s choices conflict with their own values or those of the individual’s relatives	70% agree	70% agree				
In this strategy, freedom of choice is often limited to pre-selected options, depending on the individual’s capabilities and the rules within the facility	80% agree	100% agree				
In this strategy, it is important to find a good balance between self-determination and safety, but explaining this to an individual is difficult	70% agree	100% agree				

**Table 8:** Agreement per group and rounds on advantages/disadvantages of strategy 5: Implement technology and aids.

Round	2		3		4	
**Advantages**	**Relatives**	**Professionals**	**Relatives**	**Professionals**	**Relatives**	**Professionals**
This strategy helps individuals with disabilities express desires, needs and problems clearly, making it easier for others to understand them	90% agree	70% agree				
This strategy helps individuals make choices at their own pace and provides access to a wider range of activities when the technology is tailored to an individual’s developmental level	100% agree	80% agree				
This strategy helps caregivers analyse an individual’s condition and adapt their approach accordingly, saving time and money	70% agree	10% disagree 30% neutral 60% agree		70% agree		
This strategy offers individuals a simple and effective way to maintain contact with others	10% disagree 30% neutral 60%agree	70% agree	67% agree		80% agree	
This strategy promotes individuals’ involvement in activities	40% neutral 60% agree	70% agree	89% agree			
This strategy helps an individual develop more independence, potentially lowering work pressure and increasing efficiency	40% neutral 60% agree	40% neutral 60% agree	67% agree	70% agree	70% agree	
This strategy increases an individual’s autonomy, competence and relatedness to others	40% neutral 60% agree	70% agree	89% agree			
**Disadvantages**						
One disadvantage of this strategy is that technology can be expensive and unstable, which involves practical challenges such as battery life, portability, and development and maintenance of user skills.	80% agree	90% agree				
One disadvantage of this strategy is that technology does not always align with an individual’s developmental level or specific circumstances, possibly leading to an overestimation of capabilities, fatigue and frustration.	80% agree	80% agree				
This strategy entails security and privacy risks (associated with the use of technology)	30% disagree 30% neutral 40% agree	20% disagree 20% neutral 60% agree	78% agree	50% agree 30% neutral 10% agree 10% disagree		70% agree
A disadvantage of this strategy is that not all healthcare professionals possess the required user skills for technology, plus maintaining these skills can also be hampered by time constraints, personnel changes and workload	90% agree	80% agree				
Implementing this strategy is very time-consuming for relatives and healthcare professionals	70% agree	80% agree				
This strategy may involve excessive use of technology, which may lower an individual’s motivation to use technology or engage in other activities (such as going outside)	10% disagree 40% neutral 50% agree	70% agree	67% neutral 33% agree		90% neutral	
This strategy can entail high costs that health insurers do not always reimburse	40% neutral 60% agree	70% agree	89% agree			
This strategy can lead to confusion for an individual when healthcare professionals do not use a technology uniformly	70% agree	90% agree				

**Table 9:** Agreement per group and rounds on advantages/disadvantages of strategy 6: Create and maintain a network of acquaintances around the person.

Round	2		3		4	
**Advantages**	**Relatives**	**Professionals**	**Relatives**	**Professionals**	**Relatives**	**Professionals**
This strategy enables individuals with disabilities to have new experiences and discover new interests by engaging with a wide variety of people, in this way strengthening their connection to society and enriching their living environment	90% agree	90% agree				
This strategy offers advice and support to individuals and their relatives, ensuring that healthcare professionals can take over the care duties when relatives are not available	70% agree	80% agree				
In this strategy, team members learn from each other’s knowledge and expertise, improving the quality of care and promoting an individual’s autonomy, competence and relatedness to others	90% agree	100% agree				
By incorporating the knowledge of relatives, this strategy offers healthcare professionals a more comprehensive understanding of an individual, allowing for customised care	100% agree	90% agree				
This strategy makes it easier for new employees to support an individual’s self-determination	80% agree	80% agree				
**Disadvantages**						
Implementing this strategy is difficult when there are frequent personnel changes	20% disagree 20% neutral 60% agree	70% agree	100% agree			
This strategy can shrink the network or even make it disappear as an individual becomes older or moves to a facility, leading to a loss of important knowledge about the individual	20% disagree 20% neutral 60% agree	80% agree	89% agree			
This strategy requires extensive consultation and procedures, in which the individual is not always involved	70% agree	70% agree				
In this strategy, different opinions within the network can make it difficult to arrive at a joint approach	40% disagree 10% neutral 60% agree	80% agree	89% disagree			
A disadvantage of this strategy is that creating and maintaining a network is difficult in a society that is highly individualistic (a societal culture emphasising personal freedom, independence and individual goal attainment)	30% disagree 20% neutral 50% agree	80% agree	56% disagree 45% agree		100% disagree	
This strategy is disadvantageous because differing views and historical data (such as an individual’s previous preferences) can make people cling to how things have always been done and be less open to new things and possibilities	10% disagree 40% neutral 50% agree	40% neutral 60% agree	67% agree	60% agree 30% neutral 10% disagree	100% agree	70% agree

## Results

**Fig 1:**
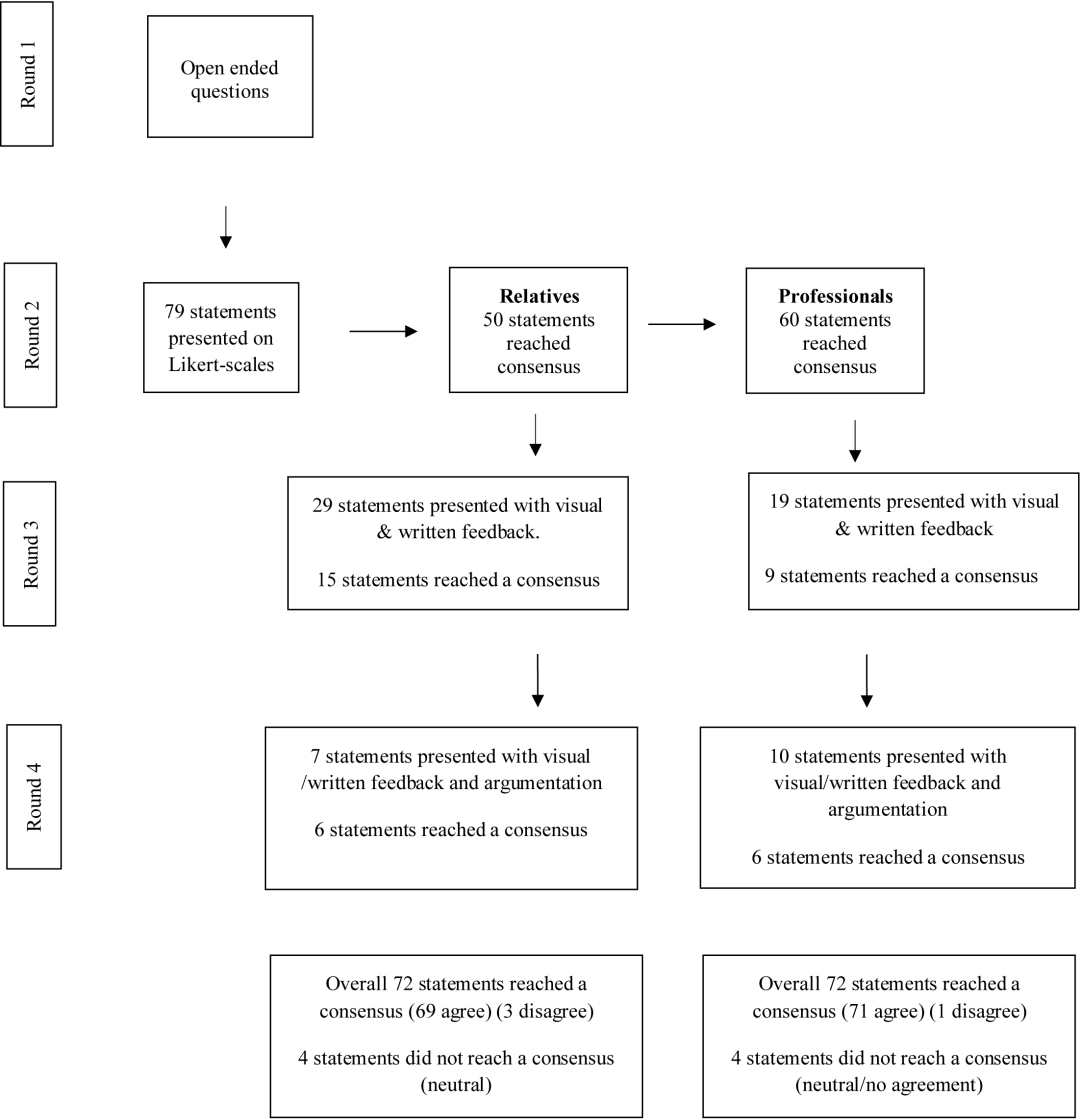
Schematic display of procedures and statements consensus per round. Figure shows procedure of the study and number of items reaching consensus per group and round.

In round one, all 13 participants together provided 619 responses. Thematic analysis of the responses from round one yielded 79 themes, which were subsequently rated for consensus in round two. These themes, along with consensus levels per participant group and per round, can be found in Tables 3 to 8. Fig 1 shows a flowchart representing how many themes reached a consensus per round. In total, no consensus was reached on 8 of the 79 rated themes. Additionally, differences emerged between the two groups regarding which themes reached consensus and in which round this occurred. These findings are presented per support strategy. For each strategy, the advantages and disadvantages that reached consensus among both groups are presented first, followed by statements where only one group reached consensus or where consensus was not reached, accompanied by participants’ arguments for disagreement.

### “Strategy 1: Equal cooperation of relatives and healthcare professionals to create an environment optimal for self-determination

#### Advantages.

Participants provided five statements regarding the advantages of Strategy 1. Four reached consensus in Round 2 and one in Round 4. Both groups agreed that this strategy promotes mutual learning between stakeholders, contributes to greater clarity and calmness for the individual, and improves support for self-determination. Participants also emphasised that cooperation benefits relatives by helping them feel involved and supported by professionals, and by preparing them for transitions in care.

#### Disadvantages.

Five disadvantage statements were identified, three of which reached consensus in Round 2. Both groups agreed that effective collaboration depends on conditions that are not always met in practice, such as stable staff, active involvement of relatives, and clear communication among those involved in the person’s care. Group differences were also observed: professionals agreed that collaboration may expose conflicting opinions and create ambiguity regarding responsibilities, whereas most relatives disagreed with these concerns in Round 2.

### Strategy 2: Build and maintain sensitivity

#### Advantages.

Participants provided five statements on the advantages of this strategy. Four reached consensus in Round 2 and one in Round 3. Both groups agreed that this strategy enhances self-expression in individuals with disabilities and enables communication partners to interpret and respond more effectively through a more coordinated team approach. Participants also noted benefits for interaction and relationships, as well as increased professional development and job satisfaction among professionals. Relatives additionally indicated that this strategy may reduce their own stress.

#### Disadvantages.

Six disadvantage statements were identified. One reached consensus in Round 2 and others in subsequent rounds. Both groups agreed that this strategy depends on conditions that are not always present in practice, including consistent interpretation of behaviour across people and settings, manageable workload, sufficient time, and stable personnel. Group differences were also found. Relatives considered this strategy time- and effort-intensive for professionals, whereas professionals disagreed, arguing that initial time investment may save time in the long term. Professionals also expressed concern that large care teams may increase the risk of divergent interpretations, while relatives remained neutral.

### Strategy 3: Purposeful application of total communication

#### Advantages.

Participants provided eight statements on the advantages of this strategy. Six reached consensus in Round 2 and one in Round 3. Both groups agreed that total communication provides structure and predictability, supports self-expression and self-esteem, and increases opportunities for individuals to influence their own lives. It was also perceived as fostering mutual understanding, reducing frustration and stress, and potentially preventing self-harming behaviours. Relatives believed this strategy could promote independence, whereas professionals remained neutral, pointing to variation in the target group’s ability to be independent.

#### Disadvantages.

Seven disadvantage statements were identified: three reached consensus in Round 2 and two in Round 3. Both groups agreed that this strategy requires appropriate and consistent use of communication aids across settings, and that implementation may be hindered by the cost of aids and associated training. Professionals additionally expressed concern that temporary discontinuation of aids, for example due to illness, could disrupt communication development, whereas relatives remained neutral. Relatives also pointed to work pressure among professionals as a barrier to implementation. Professionals did not reach consensus on this point, noting that effective communication may save time in the long run.

### Strategy 4: Support and encourage choice making

#### Advantages.

Seven statements described the advantages of this strategy, all of which reached consensus in Round 2. Participants agreed that encouraging choice making promotes autonomy, personal growth, and participation in preferred activities. It was also seen as contributing to professionals’ job satisfaction and to more client-centred care.

### Disadvantages

Four disadvantage statements reached consensus in Round 2 and one in a later round. This strategy has limited applicability, as not all individuals in the target group are able to make direct choices, and available options are often pre-selected by others and restricted to a small number of alternatives. Participants also noted the challenge of balancing choice making with health and safety concerns and with their own values. Professionals added that supporting choice making near the limits of a person’s abilities may become overly demanding and of limited value, particularly when preferences are already known. Relatives remained neutral on this point. Relatives also reported that time constraints and staff shortages can hinder implementation, whereas professionals remained neutral, suggesting that opportunities for choice making can be created through careful planning.

### Strategy 5: Implement technology and aids

#### Advantages.

Participants provided seven statements describing the advantages of this strategy. Two reached consensus in Round 2 and five in later rounds. Both groups agreed that technology can support self-expression, choice making, social contact, and participation when tailored to the individual. Technology may also help professionals analyse conditions, adapt support, and enable individuals to do things themselves, potentially saving time, reducing costs, and increasing efficiency.

#### Disadvantages.

Five disadvantage statements reached consensus in Round 2 and two in later rounds. Both groups agreed that technology may be expensive, unstable, and difficult to implement because of issues such as limited insurance reimbursement, short battery life, portability, privacy concerns, staff changes, workload, limited time, mismatch with developmental level, and inconsistent use. Professionals additionally expressed concern that overreliance on technology might reduce motivation to engage in other activities, such as going outside. Relatives remained neutral on this point, noting that some technologies are still in a pioneering phase and that a better balance may emerge over time.

### Strategy 6: Create and maintain a network of acquaintances around the person

#### Advantages.

Five advantage statements reached consensus in Round 2. Participants agreed that this strategy enables individuals to encounter new people, experiences, and interests, thereby enhancing their sense of societal connection. They also noted that a broader network facilitates the integration of relatives’ knowledge, supports a more complete understanding of the individual, and contributes to smoother transitions when professionals take over care.

### Disadvantages

Participants provided six disadvantage statements. One reached consensus in Round 2 and three more in later rounds. Both groups agreed that this strategy requires extensive consultation and coordination, during which the individual is not always actively involved. They also noted that networks of relatives may diminish over time, especially when individuals age or move into institutional care, leading to a loss of valuable personal knowledge. In addition, participants agreed that reliance on historical information may lead people to adhere to established routines and become less open to new possibilities. Professionals further noted that building and maintaining a network may be challenging in a highly individualised society. Relatives disagreed, emphasising that even when professionals change, relatives remain constant. Professionals also worried that differing views may hinder the development of a unified approach, whereas most relatives disagreed with this concern in Round 2.

## Discussion

This study aimed to explore relatives’ and healthcare professionals’ potential consensus on their perceived advantages and disadvantages of various strategies aimed at supporting self-determination in people with severe or profound intellectual and multiple disabilities. A consensus was reached on 72 statements by both groups, reflecting a substantial degree of agreement. Yet, some divergences emerged, highlighting distinct roles, experiences, and perspectives. Such differences align with earlier research showing how self-determination support is shaped by the nature of one’s relationship to the individual [21].

One of the most salient findings centered on the collaborative dynamic between relatives and professionals. Collaborative partnerships allow relatives to share crucial knowledge accumulated over years of caregiving, which can then be integrated into professional care to ensure continuity and quality. This dynamic helps relatives prepare for transitions when the primary caregiving responsibility shifts to professionals. Prior studies highlight how parents often worry about their tacit caregiving knowledge getting lost after they are no longer present, making knowledge transfer an essential aspect of collaborative support [52,53]. However, collaboration can be undermined by factors such as unclear communication channels and frequent personnel changes, preventing professionals from developing the in-depth understanding needed to provide care aligned with relative’s values and individual’s unique needs [54,52]. Given these challenges, fostering stable and communicative partnerships is imperative to maximize benefits of self-determination support.

Sensitivity to the individual’s behaviour signals emerged as another critical factor. Participants emphasized that caregivers can develop and maintain a shared sensitivity, allowing for a unified approach across caregivers in responding to the individual’s behaviour. Furthermore, healthcare professionals reported that cultivating sensitivity contributes positively to their own job satisfaction, sense of fulfilment, and the quality of their relationships with those they support [10]. Yet, developing sensitivity requires ongoing effort, training, and adequate time—resources that are frequently strained due to staffing shortages and high workloads [31]. Interestingly, professionals suggested that investing time to build sensitivity yields long-term time savings through quicker and efficient responses to behavioural cues, highlighting the need to prioritize time for professional development in this area [25].

The purposeful application of total communication, which includes personalized communication aids tailored to the individual’s abilities, was also highlighted as a foundation of autonomy as it supports communication and expression [55]. Moreover, participants noted that total communication strengthens the caregiver-individual bond, reducing frustration and stress. Such reductions are important as self-harm behaviours are unfortunately prevalent in this population, often triggered by frustration due to being misunderstood [56,57]. Simultaneously, increased caregiver responsiveness is linked to lower levels of stress and challenging behaviour [58–60]. However, inconsistent application of communication across caregivers can cause individual’s confusion, emphasizing regular communication assessments and collaboration with experts (e.g., speech therapists) to ensure appropriately tailored and cohesive communication strategies [21,61].

Supporting and encouraging choice-making was recognized as a powerful means to enhance autonomy, confidence, health, and personal growth, aligning with previous research showing correlation between choice making and improved quality of life [62]. Choice-making also opens pathways for increased societal participation according to participants. However, people within the target group often experience low levels of social participation, partly due to direct support staff focusing less on societal inclusion [63,64]. This gap points to an important area for further exploration—understanding how professionals can better facilitate societal involvement through supported choice-making. Choice making also has other limitations. Not everyone within this population can make explicit, direct choices due to communicative or cognitive challenges. Caregivers must be adept at interpreting less direct expressions of preference to honour the individual’s wishes [7]. Ethical dilemmas were also mentioned, such as when individuals choose actions that may pose risks—like refusing appropriate clothing or engaging in potentially unsafe activities. Caregivers often struggle to balance respecting autonomy with ensuring safety and health, a challenge well documented in caregiving literature [65].

Technology’s role in supporting self-determination was viewed positively by participants, who noted that various devices can positively influence autonomy, activity participation, self-expression, and social contact [55]. Technology also aids caregivers in monitoring individual’s physical and mental condition, providing objective data to inform care decisions. This capability is particularly valuable during transitions, such as when individuals transit between caregivers or settings [66,67]. However, these benefits may be undermined by inconsistent technology use across care settings. For instance, when equipment is not transferred between residence and day-care settings due to limited portability, short battery life, and communication barriers between professionals [21].

Creating and maintaining a network of acquaintances and social contacts around individuals was identified as an important advantage for promoting participation and connection to society. Participants shared stories illustrating how exposure to different cultures, introduced by professionals from diverse backgrounds, allowed individuals to experience new foods, music, and customs, opportunities that might otherwise be unavailable to them. One participant described how attending church with a new professional enabled an individual to build relationships and become part of a new community. Exposures like these may enrich the target group’s experience of the world and foster their social inclusion [21,68]. According to participants, networks can also provide professionals access to family knowledge. In turn, relatives may feel more confident in professionals’ ability to care appropriately for the individual in their absence. This suggests that strong collaborative networks may help address relatives’ concerns about professionals’ ability to provide adequate support [15,52]. However, participants also noted that networks can deteriorate over time, particularly as individuals age and move into institutional care—an environment associated with reduced informal contact [11,64]. Contributing factors include aging relatives and staff turnover, challenging sustained engagement. Professionals observed broader societal trends toward individualism where people are increasingly busy with personal obligations, reducing network building. Relatives continued to view themselves as stable, enduring network members, a perspective aligning with prior findings [15].

The differences between relatives and healthcare professionals in how they evaluated the advantages and disadvantages of self-determination support strategies may partly reflect differences in role, knowledge base, and positional influence within support contexts [21]. Relatives often draw on long-term, relational, and tacit knowledge of the person, whereas professionals may rely more strongly on formal training, organisational routines, and considerations of feasibility, accountability, and risk [21]. These differing epistemological positions may help explain why relatives more often emphasised continuity, familiarity, and practical barriers such as staff shortages, while professionals were more likely to raise concerns about conflicting interpretations, division of responsibilities, and the conditions needed for consistent implementation.

In addition, the two groups operate in different care contexts and under different constraints. For relatives living with the person, support is embedded in everyday family life, whereas relatives of individuals in residential settings may take on a more consultative or advocacy-oriented roles [15,21]. Professionals, in turn, often work within institutional structures that shape what is feasible in practice through time limitations, staffing patterns, organisational policies, and accountability requirements [69]. These contextual differences may also create unequal influence over how support strategies are enacted: professionals may have greater formal control over implementation in daily care, while relatives often provide continuity of knowledge across changing care situations. Taken together, these differences in role, knowledge, and institutional positioning may help explain why the two groups did not always evaluate the same strategies in the same way.

### Strengths and limitations

A key strength of this study lies in the inclusion of a diverse group of relatives and professionals who represented various roles and occupational backgrounds, enhancing the validity of the findings. Additionally, the use of the Delphi method allowed stakeholders to express their views independently, without the influence of group dynamics [47]. Nonetheless, several limitations should be noted. The study involved a relatively small sample (23 participants), which may limit the generalizability of the findings. However, previous research shows that Delphi studies with similar sample sizes produce reliable results [70], and methodological guidelines suggest that panels of around 10 participants per group are generally sufficient [39].

Additionally, as the study was conducted in the Netherlands, the transferability of the findings to other countries requires consideration. There is considerable variation in how disability services are organised and implemented across countries [23]. Differences exist not only between geographically distant countries operating under distinct legal, financial, and service delivery systems, but also among countries that formally align their policies with shared frameworks such as the United Nations Convention on the Rights of Persons with Disabilities. Furthermore, variation in the implementation of policies regarding access to assistive technologies, supported decision-making, inclusive education, and the adaptation of disability care to individual needs is observed even among geographically proximate countries operating under common regulatory structures, such as within the European Union [23,71,72].

These structural and cultural differences may influence the roles of family members and professionals, the availability of resources, and the extent to which person-centred and autonomy-supportive practices are embedded in daily care. As this study was conducted within the Dutch disability service system, characterised by relatively well-developed formal support structures [22], the applicability of these findings may differ in contexts where services are primarily family-based, differently organised, or more limited in resources. These contextual differences may influence how self-determination is conceptualised, prioritised, and operationalised in daily practice. Consequently, a limitation of this study is the restricted transferability of its findings to settings with substantially different cultural norms or service systems.

Another limitation of this study concerns the operationalisation of participants’ expertise. Although participants were selected based on sustained practical involvement with the target population, no formal or standardised criteria were used to validate or stratify their level of expertise. While this approach aligns with previous Delphi research [33], it may have resulted in variability in the depth and type of expertise within the panel, which could have influenced the nature of the consensus achieved. Previous research indicates that factors such as occupational role and length of experience in communicating with individuals from the target group shape how professionals conceptualise and describe their support for self-determination [21]. It is therefore possible that participants with more extensive experience were better able to articulate a broader and more nuanced range of advantages and disadvantages in the earlier rounds, whereas participants with less experience may have been more likely to engage with perspectives introduced by others in later rounds. As such, the consensus reached may not always reflect uniformly grounded expertise, but rather the convergence of perspectives shaped by differing forms and levels of experience. An additional limitation concerns the reliance on relatives and professionals to report on the advantages and disadvantages of support strategies. While their interpretations provide valuable practical insights, the findings reflect their perspectives and may not fully capture the subjective experiences of support recipients themselves on advantages and disadvantages of support strategies. As such, the results should be interpreted as stakeholder perceptions rather than direct evidence of impact on the persons with disabilities. The use of interviews in the first round may have given greater weight to participants who are more verbally articulate, potentially limiting the extent to which the perspectives of participants with more limited verbal expression were equally represented. Lastly, while the Delphi method is thought to reduce conformity pressure, consensus in rounds 3 and 4 may have been affected by participants’ tendency to conform to the majority [36,73], especially as themes were not reformulated between rounds and participants were asked to explain their answers.

### Implications for future research

To our knowledge, the effectiveness of current support strategies remains difficult to test, largely because it remains unclear how self-determination in people with severe or profound intellectual and multiple disabilities should be measured [25,26]. This study aimed to provide insights into the perceived advantages of such strategies from the perspective of those who apply them. To interpret the findings, we integrated insights from Self-Determination Theory (SDT) [24] and Causal Agency Theory (CAT) [27,74], thereby considering both the psychological needs underlying self-determination and the ways in which these needs may be expressed behaviourally and responded to by the social environment.

In SDT, autonomy refers to experiencing one’s behaviour as volitional and aligned with one’s own preferences and values. Strategies such as supporting choice making, using total communication, and applying assistive technology may facilitate the expression of preferences and intentions, thereby supporting autonomy, particularly when individuals have limited verbal communication [55]. Cooperation between relatives and healthcare professionals may further support autonomy by improving the consistency with which preferences and behaviours are interpreted and responded to [25]. At the same time, CAT highlights that these strategies may also strengthen the individual’s capacity to act as a causal agent by increasing the likelihood that their behaviour has meaningful effects in the environment.

However, the present findings also suggest that existing theoretical assumptions may require refinement when applied to this population. In individuals with severe or profound intellectual and multiple disabilities, preferences and intentions are often not directly accessible and are frequently inferred through behaviour and interpreted by others. This means that autonomy cannot always be understood solely as an internally experienced or independently expressed phenomenon. Rather, our findings suggest that autonomy may need to be conceptualised as a relational and interpretative process, in which the accurate recognition of preferences depends on sensitive, responsive, and coordinated support. In this sense, the findings extend SDT by highlighting the interpersonal and contextual conditions under which autonomy-supportive practice becomes possible when preferences cannot be expressed symbolically or independently.

Competence, defined as the experience of effectiveness in interacting with the environment, may be supported when individuals receive sensitive and attuned responses to their communicative attempts [7,19]. When behaviour reliably leads to meaningful environmental responses, individuals may develop expectations of efficacy, which aligns with CAT’s emphasis on causal agency [27,74]. Maintaining a broad social network may primarily support relatedness by promoting social inclusion and meaningful interpersonal connections [63]. At the same time, from an integrated SDT–CAT perspective, such relationships also form the interpersonal context in which causal agency can be realised, as supportive others are needed to recognise and respond to the person’s actions. Taken together, the findings suggest that the value of support strategies lies not only in their potential to support autonomy, competence, and relatedness, but also in the extent to which they enable individuals to act as causal agents through observable interaction with a responsive environment

Given the small sample size (*n* = 23), formal statistical analyses examining age-, experience-, or gender-related differences would be underpowered and potentially unreliable. Therefore, no generalizing statements are made regarding demographic differences. Nevertheless, a tentative pattern was observed in the qualitative data from the first Delphi round. While participants across age groups emphasised the importance of long-standing knowledge of the person, some of the statements concerning potential limitations of relying predominantly on historical knowledge were initiated by relatively younger relatives and healthcare professionals. These participants highlighted that individuals with severe or profound intellectual and multiple disabilities continue to develop over time, with emerging behaviours, preferences, and interests requiring ongoing recognition. Similarly, several statements advocating for broader support networks and exposure to new people and experiences were introduced by younger participants. These observations are exploratory and based on the origin of statements rather than on statistically tested subgroup differences in agreement levels. As such, they should be interpreted with caution. However, they may suggest potential generational differences in emphasis, warranting further investigation in larger and more diverse samples.

Not only is it important to include larger samples providing stronger evidence of associations, but also to study if stakeholder’s perspectives reflect the perspectives of the person with disabilities. Future studies should move beyond correlational analyses and proxy reporting by incorporating inclusive methods aiming to capture the perspectives of individuals with disabilities [75,76]. Inclusive approaches often use ethnographic methods to incorporate the experiences of individuals through interpretation of informed proxies—such as relatives or professionals—who can attempt to articulate the lived realities of the person [7,75]. This relational autonomy, positions proxies as essential communicators of the individual’s experiences, making them a vital component in inclusive research [75]. Although such methodologies are still evolving, they hold significant promise and merit further exploration. Future research could also explore whether insights into the advantages and disadvantages of support strategies can aid decision-making in practice. Although people with severe or profound intellectual and multiple disabilities share certain characteristics, they are highly heterogeneous in their abilities and needs [37], which may lead to individual differences in how these strategies are experienced. Further studies could examine how relatives and professionals can be supported in assessing which strategies are likely to be beneficial or burdensome for a specific individual, thereby improving tailored decision-making. Ahmadi and colleagues [77] conducted a Delphi study to examine which teacher behaviours were most strongly associated with support for autonomy, competence, and relatedness. Based on expert consensus, they proposed a classification system of motivational behaviours that provides concrete behavioural descriptions of how these psychological needs may be supported or hindered in practice. Although developed in an educational context, this approach offers a useful methodological example for future research on individuals with severe or profound intellectual and multiple disabilities. Future studies could adapt a similar classification approach to examine which support behaviours of relatives and healthcare professionals are most likely to foster autonomy, competence, and relatedness in this population. This may help strengthen the connection between theory and practice and contribute to a more precise operationalisation of self-determination in everyday support contexts.

### Implications for practice

The results of this study offer several implications for practice and policy by identifying conditions that may help relatives and healthcare professionals to sustain support for self-determination in everyday care. Across strategies, the perceived benefits were often accompanied by barriers or preconditions, suggesting that the successful use of support strategies depends not only on their content, but also on the organisational and relational conditions under which they are implemented.

First, participants indicated that technology and assistive communication aids may help individuals express needs and preferences, support choice making, and assist professionals in assessing and responding to the individual’s condition. However, these benefits were often limited by practical barriers such as insufficient funding, inconsistent use across settings, and lack of continuity between caregivers. To address this, organisations could implement shared care plans that specify how communication aids should be used across home, residential, and day-care contexts, supported by regular multidisciplinary meetings and clearly defined responsibilities. The use of shared reporting formats or accessible digital communication systems may further improve continuity between relatives and professionals [21].

Second, difficulties in collaboration between relatives and professionals suggest a need for more explicit structures for knowledge exchange. As individuals often grow up in family contexts where self-determination is supported through relational and experiential knowledge [1,15], and later move into services shaped by professional routines and organisational values [20], effective support depends on integrating these different forms of expertise [21]. Practice and policy should therefore prioritise conditions that facilitate collaboration, including protected time for consultation, formal expectations for family involvement in care planning, and training for professionals in partnership-oriented working [21,77,78].

Several existing initiatives may support such implementation. For example, the ‘Skills for Growing Up–Profound Intellectual and Multiple Disabilities’ (SGU-PIMD) tool developed by Luitwieler and colleagues [29] could be embedded in routine transition planning to support communication among young people, parents, and professionals. Similarly, the structured group dialogue described by Kruithof and colleagues [79] could be incorporated into periodic care evaluation meetings to support discussion about future caregiving roles. Additional evidence-based programmes used in the Netherlands and internationally [44,45] should also be systematically evaluated and, where appropriate, adapted for broader implementation in adult services.

Third, participants identified time pressure, staff shortages, and personnel turnover as major barriers to the use of self-determination support strategies. This suggests that practice-level improvements alone are unlikely to be sufficient without organisational and policy support. Organisations may benefit from staffing policies that prioritise continuity of care, such as maintaining small and stable teams and reducing frequent staff reassignments [52]. In addition, previous research highlights the importance of participatory management, supervisor support, professional development opportunities, manageable workloads, fair compensation, and realistic job expectations for staff retention and person-centred service delivery [80]. At the policy level, these findings point to the need for sustained funding, workforce planning, and investment in training and onboarding procedures to ensure that self-determination support can be implemented consistently over time [79–81].

## Conclusion

This study highlights a recurring tension in the support of individuals with severe or profound intellectual and multiple disabilities: the need to balance structured strategies (such as standardized communication aids and formal collaboration) with relational flexibility, including sensitive intuitive action. This tension reflects a broader challenge in care systems; ensuring stability while leaving space for adaptive learning and human connection [21]. Policy could adopt a dual focus: investing in professional frameworks that offer consistent resources, while empowering support network members to build relationships with care recipients and with each other. Self-determination for people with severe or profound intellectual and multiple disabilities does not occur in isolation, it depends on coordinated efforts among relatives, professionals, and broader networks. Support strategies are most effective when enacted collaboratively, thus self-determination is not solely an individual trait but a shared responsibility within the support network [7,19]. Therefore, further research should focus on identifying practical solutions to the barriers experienced by families and professionals in their collaborate efforts to support self-determination.

## Supporting information

S1 File Overview of Strategies for Supporting Self-Determination in Individuals with Severe or Profound Intellectual and Multiple Disabilities: Pre-Delphi Process Compilation. This supplementary file presents an overview of strategies to support self-determination in individuals with severe or profound intellectual and multiple disabilities, compiled prior to the Delphi process. The overview is based on a systematic review, concept mapping study, prior Delphi study, and Dutch healthcare practice literature, with emphasis on empirically supported interventions.
(DOCX)

## References

[1] Mumbardó-Adam C, Arellano A, Vicente E, Berástegui A. How should we support families of people with intellectual disability to promote their self-determination? A Delphi study on critical components for intervention. J Intellect Dev Disabil. 2023;48(4):357–69. doi: 10.3109/13668250.2023.2234547 39815883

[2] Beadle-Brown J, Beecham J, Leigh J, Whelton R, Richardson L. Outcomes and costs of skilled support for people with severe or profound intellectual disability and complex needs. J Appl Res Intellect Disabil. 2021;34(1):42–54. doi: 10.1111/jar.12782 32755061

[3] Elliott C, Dillenburger K. The effect of choice on motivation for young children on the autism spectrum during discrete trial teaching. Research in Spec Educ Needs. 2014;16(3):187–98. doi: 10.1111/1471-3802.12073

[4] Sullivan WE, Roane HS. Incorporating choice in differential reinforcement of other behavior arrangements. Behavioral Development. 2018;23(2):130–7. doi: 10.1037/bdb0000079

[5] Vaucher C, Cudré-Mauroux A, Piérart G. Environmental, Personal, and Relational Barriers and Facilitators to Self-Determination among Adults with Intellectual Disabilities. Scandinavian Journal of Disability Research. 2020;22(1):97–107. doi: 10.16993/sjdr.624

[6] Watson J. Stretching beyond our perceived boundaries: The role of speech-language pathology in realising autonomy through supported decision-making. Int J Speech Lang Pathol. 2023;25(3):355–62. doi: 10.1080/17549507.2023.2187331 37038630

[7] Skarsaune SN, Hanisch HM. Holding and professional care: On self-determination for persons with profound intellectual and multiple disabilities. Research and Practice for Persons with Severe Disabilities. 2023;48(1):25–40. doi: 10.1177/15407969231153579

[8] United Nations. Convention on the rights of persons with disabilities (CRPD). https://www.un.org/development/desa/disabilities/convention-on-the-rights-of-persons-with-disabilities.html. 2006. Accessed 2023 October 1.

[9] Zaks Z. Changing the medical model of disability to the normalization model of disability: clarifying the past to create a new future direction. Disability & Society. 2023;39(12):3233–60. doi: 10.1080/09687599.2023.2255926

[10] Nieuwenhuijse AM, Willems DL, van Goudoever JB, Olsman E. The perspectives of professional caregivers on quality of life of persons with profound intellectual and multiple disabilities: a qualitative study. Int J Dev Disabil. 2020;68(2):190–7. doi: 10.1080/20473869.2020.1737469 35309693 PMC8928810

[11] Kamstra A, van der Putten AAJ, Vlaskamp C. The structure of informal social networks of persons with profound intellectual and multiple disabilities. J Appl Res Intellect Disabil. 2015;28(3):249–56. doi: 10.1111/jar.12134 25431193

[12] Hostyn I, Maes B. Interaction between persons with profound intellectual and multiple disabilities and their partners: a literature review. J Intellect Dev Disabil. 2009;34(4):296–312. doi: 10.3109/13668250903285648 19903121

[13] Nind M, Strnadová I. Pushing the boundaries of inclusion. London: Routledge. 2020.

[14] Maes B, Nijs S, Vandesande S, Van Keer I, Arthur-Kelly M, Dind J, et al. Looking back, looking forward: Methodological challenges and future directions in research on persons with profound intellectual and multiple disabilities. J Appl Res Intellect Disabil. 2021;34(1):250–62. doi: 10.1111/jar.12803 33073444

[15] Kruithof K, Willems D, van Etten-Jamaludin F, Olsman E. Parents’ knowledge of their child with profound intellectual and multiple disabilities: An interpretative synthesis. J Appl Res Intellect Disabil. 2020;33(6):1141–50. doi: 10.1111/jar.12740 32367663 PMC7687241

[16] Dorsman NI, Waninge A, van der Schans CP, Luijkx J, Van der Putten AAJ. The roles of adult siblings of individuals with a profound intellectual disability. J Appl Res Intellect Disabil. 2023;36(6):1308–18. doi: 10.1111/jar.13149 37550062

[17] Nguyen L, Jack SM, Davis H, Bellefeuille S, Arafeh D, Di Rezze B. Being a sibling of a youth with a neurodisability: A qualitative study about the roles and responsibilities during the transition to adulthood. Child: care, health and development. 2024;50(2):e13241. doi: 10.1111/cch.13241 38445673

[18] van Tuyll van Serooskerken JM, Willemen AM, de la Croix A, Embregts PJCM, Schuengel C. Satisfying Basic Psychological Needs among People with Complex Support Needs: A Self-Determination Theory-Guided Analysis of Primary Relatives’ Perspectives. Disabilities. 2022;2(2):330–47. doi: 10.3390/disabilities2020024

[19] Hanisch H, Skarsaune SKN. Rethinking empathy: professional work with persons with PIMD. Med Humanit. 2024;50(3):570–80. doi: 10.1136/medhum-2023-012783 38937088

[20] Kuld PB, Frielink N, Zijlmans M, Schuengel C, Embregts PJCM. Promoting self-determination of persons with severe or profound intellectual disabilities: a systematic review and meta-analysis. J Intellect Disabil Res. 2023;67(7):589–629. doi: 10.1111/jir.13036 37165964

[21] Kúld PB, Frielink N, Schuengel C, Embregts PJCM. Supporting self-determination of individuals with severe or profound intellectual and multiple disabilities according to relatives and healthcare professionals: A concept mapping study. J Appl Res Intellect Disabil. 2024;37(4):e13267. doi: 10.1111/jar.13267 38863165

[22] Hauwert S. Ruimte voor eigen regie bij mensen met ernstige meervoudige beperkingen. 2018. https://disabilitystudies.nl/sites/default/files/proefschrift_ruimte_voor_eigen_regie_bij_mensen_met_emb.pdf

[23] Verdugo MA, Jenaro C, Calvo I, Navas P. Disability Policy Implementation From a Cross-Cultural Perspective. Intellect Dev Disabil. 2017;55(4):234–46. doi: 10.1352/1934-9556-55.4.234 28753399

[24] Ryan RM, Deci EL. Self-determination theory: basic psychological needs in motivation, development, and wellness. New York: Guilford Press. 2017.

[25] Nijs S, Zijlmans M, Schuengel C, Embregts PJCM. Operationalisation of self-determination of persons with profound intellectual and multiple disabilities: A Delphi study. J Intellect Dev Disabil. 2023;48(3):300–12. doi: 10.3109/13668250.2022.2147053 39815922

[26] van Tuyll van Serooskerken JM, Willemen AM, Embregts PJ, Schuengel C. Change in self-determination-related constructs in persons with severe or profound intellectual and multiple disabilities in the context of transitions. J Intellect Disabil. 2025;:17446295251317759. doi: 10.1177/17446295251317759 39885634

[27] Shogren KA, Wehmeyer ML, Palmer SB, Forber-Pratt AJ, Little TJ, Lopez S. Causal agency theory: reconceptualizing a functional model of self-determination. Education and Training in Autism and Developmental Disabilities. 2015;50(3):251–63. doi: 10.1007/978-3-031-04260-7_14

[28] Finlay WML, Antaki C, Walton C. Saying no to the staff: an analysis of refusals in a home for people with severe communication difficulties. Sociol Health Illn. 2008;30(1):55–75. doi: 10.1111/j.1467-9566.2007.01028.x 18254833

[29] Luitwieler N, Luijkx J, van der Stege HA, Grootoonk A, van der Schans CP, van der Putten AAJ, et al. Transition to adulthood of adolescents with profound intellectual and multiple disabilities: Content validation of the SGU-PIMD to support families. J Appl Res Intellect Disabil. 2024;37(1):e13161. doi: 10.1111/jar.13161 37793995

[30] Watson J, Voss H, Bloomer MJ. Placing the preferences of people with profound intellectual and multiple disabilities at the center of end-of-life decision making through storytelling. Research and Practice for Persons with Severe Disabilities. 2019;44(4). doi: 10.1177/1540796919879701

[31] Nicholson C, Finlay WML, Stagg S. Self-determination and co-operation in supported mealtimes involving people with severe intellectual disabilities. Disabil Rehabil. 2023;45(17):2741–50. doi: 10.1080/09638288.2022.2104941 36005211

[32] Nieuwenhuijse AM, Willems DL, van Goudoever JB, Olsman E. Parent perspectives on the assessment of quality of life of their children with profound intellectual and multiple disabilities in the Netherlands. Res Dev Disabil. 2023;139:104536. doi: 10.1016/j.ridd.2023.104536 37269577

[33] Niederberger M, Schifano J, Deckert S, Hirt J, Homberg A, Köberich S, et al. Delphi studies in social and health sciences-Recommendations for an interdisciplinary standardized reporting (DELPHISTAR). Results of a Delphi study. PLoS One. 2024;19(8):e0304651. doi: 10.1371/journal.pone.0304651 39186713 PMC11346927

[34] Habibi SA, Sarafrazi A, Izadyar S. Delphi technique theoretical framework in qualitative research. Int J Eng Sci. 2014;3(4):8–13.

[35] Powell C. The Delphi technique: myths and realities. J Adv Nurs. 2003;41(4):376–82. doi: 10.1046/j.1365-2648.2003.02537.x 12581103

[36] de Wit W, Frielink N, Roeg D, Embregts PJCM. Sexual support and education for adults with mild intellectual disabilities: a Delphi study on multiple perspectives. J Intellect Disabil Res. 2024;68(11):1267–86. doi: 10.1111/jir.13172 39021295

[37] Nouwens PJG, Smulders NBM, Embregts PJCM, van Nieuwenhuizen C. Differentiating care for persons with mild intellectual disability or borderline intellectual functioning: a Delphi study on the opinions of primary and professional caregivers and scientists. BMC Psychiatry. 2020;20(1):57. doi: 10.1186/s12888-020-2437-4 32039715 PMC7008567

[38] Wessels MD, Paap MCS, van der Putten AAJ. The content validity of the Behavioural Appraisal Scales in people with profound intellectual and multiple disabilities: A Delphi study. Policy Practice Intel Disabi. 2022;19(1):86–101. doi: 10.1111/jppi.12409

[39] Brady SR. Utilizing and Adapting the Delphi Method for Use in Qualitative Research. International Journal of Qualitative Methods. 2015;14(5). doi: 10.1177/1609406915621381

[40] Taggart L, Marriott A, Cooper M, Atkinson D, Griffiths L, Ward C, et al. Developing curricular-content and systems-related impact indicators for intellectual disability awareness training for acute hospital settings: A modified International Delphi Survey. J Adv Nurs. 2022;78(7):2055–74. doi: 10.1111/jan.15123 34866230

[41] Nakken H, Vlaskamp C. A Need for a Taxonomy for Profound Intellectual and Multiple Disabilities. Policy Practice Intel Disabi. 2007;4(2):83–7. doi: 10.1111/j.1741-1130.2007.00104.x

[42] Boat TF, Wu JT. Mental disorders and disabilities among low-income children. Washington DC: National Academies Press. 2015. doi: 10.17226/21780 26632628

[43] Orenstein GA, Lewis L. Erikson’s stages of psychosocial development. StatPearls. Treasure Island (FL): StatPearls Publishing. 2022. 32310556

[44] Dekker A, IJpma I, Martens M. De grote methodiekengids: definitie, inventarisatie, praktische toepasbaarheid en wetenschappelijke onderbouwing van begeleidingsmethodieken voor mensen met een verstandelijke beperking. Groningen: University of Groningen Press. 2024.

[45] Vilans. Vilans erkende methodes: databank interventies. https://www.databankinterventies.nl/. 2025.

[46] Hsu CC, Sandford B. The Delphi technique: making sense of consensus. Practical Assessment, Research and Evaluation. 2007;12(1):10. doi: 10.7275/pdz9-th90

[47] R Avella J. Delphi Panels: Research Design, Procedures, Advantages, and Challenges. IJDS. 2016;11:305–21. doi: 10.28945/3561

[48] Braun V, Clarke V. Thematic analysis: a practical guide. London: Sage Publications. 2022.

[49] ATLAS.ti qualitative data analysis software. Berlin: Scientific Software Development GmbH. 2023.

[50] ATLAS.ti Scientific Software Development GmbH. ATLAS.ti. Berlin: ATLAS.ti Scientific Software Development GmbH. 2023.

[51] IBM Corp. IBM SPSS Statistics for Windows. Armonk (NY): IBM Corp. 2022.

[52] Kruithof K, Olsman E, Nieuwenhuijse A, Willems D. “I hope I’ll outlive him”: A qualitative study of parents’ concerns about being outlived by their child with profound intellectual and multiple disabilities. J Intellect Dev Disabil. 2022;47(2):107–17. doi: 10.3109/13668250.2021.1920377 39818581

[53] Martínez-Tur V, Estreder Y, Moliner C, Gracia E, Pătraș L, Zornoza A. Dialogue between workers and family members is related to their attitudes towards self-determination of individuals with intellectual disability. Journal of Intellectual & Developmental Disability. 2018;43(3):370–9. doi: 10.3109/13668250.2017.1416256

[54] Hoogsteyns M, Zaal-Schuller I, Huisman S, Nieuwenhuijse AM, van Etten-Jamaludi F, Willems D, et al. Tacit knowledge in dyads of persons with profound intellectual and multiple disabilities and their caregivers: An interpretative literature study. J Appl Res Intellect Disabil. 2023;36(5):966–77. doi: 10.1111/jar.13134 37339925

[55] Lancioni GE, Singh NN, Sigafoos J, O’Reilly MF, Oliva D. Technology-Aided Programs for Persons with Severe/Profound and Multiple Disabilities: A Selective Review. Comprehensive Psychology. 2012;2:07.IT.1.1. doi: 10.2466/07.it.1.1

[56] Poppes P, van der Putten AJJ, Vlaskamp C. Frequency and severity of challenging behaviour in people with profound intellectual and multiple disabilities. Res Dev Disabil. 2010;31(6):1269–75. doi: 10.1016/j.ridd.2010.07.017 20728304

[57] Poppes P, van der Putten A, Post W, Frans N, Ten Brug A, van Es A, et al. Relabelling behaviour. The effects of psycho-education on the perceived severity and causes of challenging behaviour in people with profound intellectual and multiple disabilities. J Intellect Disabil Res. 2016;60(12):1140–52. doi: 10.1111/jir.12299 27189898

[58] Doodeman TWM, Schuengel C, Sterkenburg PS. Effects of the Attune & Stimulate-checklist for caregivers of people with severe and profound intellectual disabilities: A randomised controlled trial. J Appl Res Intellect Disabil. 2023;36(5):1136–49. doi: 10.1111/jar.13135 37365773

[59] Oliver C, Ellis K, Agar G, Bissell S, Cheuk J, Crawford H, et al. Distress and challenging behavior in people with profound or severe intellectual disability and complex needs: assessment of causes and evaluation of intervention outcomes. International Review of Research in Developmental Disabilities. 2022:109–89. doi: 10.1016/bs.irrdd.2022.05.004

[60] Sterkenburg P, Schuengel C, Janssen C. Developing a therapeutic relationship with a blind client with a severe intellectual disability and persistent challenging behaviour. Disabil Rehabil. 2008;30(17):1318–27. doi: 10.1080/09638280701482597 17852277

[61] Chadwick D, Buell S, Goldbart J. Approaches to communication assessment with children and adults with profound intellectual and multiple disabilities. J Appl Res Intellect Disabil. 2019;32(2):336–58. doi: 10.1111/jar.12530 30430716 PMC7379986

[62] Petry K, Maes B, Vlaskamp C. Domains of Quality of Life of People with Profound Multiple Disabilities: the Perspective of Parents and Direct Support Staff. Research Intellect Disabil. 2005;18(1):35–46. doi: 10.1111/j.1468-3148.2004.00209.x

[63] Hanzen G, van Nispen RMA, Vlaskamp C, Korevaar EL, Waninge A, van der Putten AAJ. Improving the participation of adults with visual and severe or profound intellectual disabilities: a process evaluation of a new intervention. BMC Health Serv Res. 2020;20(1):319. doi: 10.1186/s12913-020-05161-1 32299453 PMC7164344

[64] Kamstra A, van der Putten AA, Vlaskamp C. Efforts to increase social contact in persons with profound intellectual and multiple disabilities: Analysing individual support plans in the Netherlands. J Intellect Disabil. 2017;21(2):158–74. doi: 10.1177/1744629516653037 27283285

[65] Peterson A, Karlawish J, Largent E. Supported Decision Making With People at the Margins of Autonomy. Am J Bioeth. 2021;21(11):4–18. doi: 10.1080/15265161.2020.1863507 33372858 PMC8239054

[66] Crowe B, Machalicek W, Wei Q, Drew C, Ganz J. Augmentative and Alternative Communication for Children with Intellectual and Developmental Disability: A Mega-Review of the Literature. J Dev Phys Disabil. 2022;34(1):1–42. doi: 10.1007/s10882-021-09790-0 33814873 PMC8009928

[67] Engelhardt M, Kosiedowski M, Duszyńska I. Assistive technology for people with PIMD in challenging scenarios. JET. 2020;14(2):87–97. doi: 10.1108/jet-12-2019-0056

[68] Svanelöv E, Stier J, Enarsson P, Wallén EF, Talman L. Addressing Participation and Intellectual Disability: A Discourse Analysis of Rhetoric from Social Support Staff and Disability Sports Leaders. Scandinavian Journal of Disability Research. 2024;26(1):650–69. doi: 10.16993/sjdr.1167

[69] Hanzen G, Waninge A, Vlaskamp C, van Nispen RMA, van der Putten AAJ. Participation of adults with visual and severe or profound intellectual disabilities: Analysis of individual support plans. Res Dev Disabil. 2018;83:132–41. doi: 10.1016/j.ridd.2018.08.012 30196028

[70] Akins RB, Tolson H, Cole BR. Stability of response characteristics of a Delphi panel: application of bootstrap data expansion. BMC Med Res Methodol. 2005;5:37. doi: 10.1186/1471-2288-5-37 16321161 PMC1318466

[71] Borg J, Lindström A, Larsson S. Assistive technology in developing countries: a review from the perspective of the Convention on the Rights of Persons with Disabilities. Prosthet Orthot Int. 2011;35(1):20–9. doi: 10.1177/0309364610389351 21515886

[72] Bratan T, Fischer P, Maia M, Aschmann V. Implementation of the UN Convention on the Rights of Persons with Disabilities: A Comparison of Four European Countries with Regards to Assistive Technologies. Societies. 2020;10(4):74. doi: 10.3390/soc10040074

[73] Diamond IR, Grant RC, Feldman BM, Pencharz PB, Ling SC, Moore AM, et al. Defining consensus: a systematic review recommends methodologic criteria for reporting of Delphi studies. J Clin Epidemiol. 2014;67(4):401–9. doi: 10.1016/j.jclinepi.2013.12.002 24581294

[74] Shogren KA, Raley SK. Causal agency theory: a theoretical framework for understanding self-determination. In: Shogren KA, Raley SK, editors. Self-determination and causal agency theory. Cham: Springer. 2022. p. 49–68.

[75] de Haas C, Grace J, Hope J, Nind M. Doing Research Inclusively: Understanding What It Means to Do Research with and Alongside People with Profound Intellectual Disabilities. Social Sciences. 2022;11(4):159. doi: 10.3390/socsci11040159

[76] Penninga W, Hendriks AHC, van Bakel HJA, Embregts PJCM. Situational experiences of meaningfulness of support staff during their interactions with people with profound intellectual disabilities: An explorative study. J Intellect Dev Disabil. 2025;50(3):366–79. doi: 10.3109/13668250.2024.2447998 39946744

[77] Ahmadi A, Noetel M, Parker P, Ryan RM, Ntoumanis N, Reeve J, et al. A classification system for teachers’ motivational behaviors recommended in self-determination theory interventions. J Educ Psychol. 2023;115(8):1158–76. doi: 10.1037/edu0000783

[78] van Beurden K, Vereijken FR, Frielink N, Embregts PJCM. The needs of family members of people with severe or profound intellectual disabilities when collaborating with healthcare professionals: a systematic review. J Intellect Disabil Res. 2025;69(1):1–29. doi: 10.1111/jir.13199 39569759 PMC11621588

[79] Kruithof K, Olsman E, Willems D, Volkers K, Kleijwegt B, Nieuwenhuijse A. “What if I’m no longer around?”: An evaluative description of a structured group conversation about the care for persons with profound intellectual and multiple disabilities when they outlive their parents. J Appl Res Intellect Disabil. 2024;37(2):e13185. doi: 10.1111/jar.13185 38097399

[80] Johnson KE, Bailey CE, Weiss NR, Eidelman SM. Direct Support Professionals’ Perspectives on Workplace Support: Underappreciated, Overworked, Stressed Out, and Stretched Thin. Intellect Dev Disabil. 2021;59(3):204–16. doi: 10.1352/1934-9556-59.3.204 34030180

[81] Peters V, Frielink N, van Leest C, Heerkens L, Embregts P. Impact pathways: putting workers front and center in addressing workforce shortages in intellectual disability care. IJOPM. 2024;44(13):251–62. doi: 10.1108/ijopm-02-2024-0086

