# Supplementary material for: Advantages and disadvantages of self-determination support strategies for people with severe or profound intellectual and multiple disabilities: A Delphi study
Source: PLoS One. 2026 May 19;21(5):e0349403. doi: 10.1371/journal.pone.0349403 (PMC13186378; doi:10.1371/journal.pone.0349403)
Supplement: S1 File — This supplementary file presents an overview of strategies to support self-determination in individuals with severe or profound intellectual and multiple disabilities, compiled prior to the Delphi process. The overview is based on a systematic review, concept mapping study, prior Delphi study, and Dutch healthcare practice literature, with emphasis on empirically supported interventions. (DOCX) [file pone.0349403.s001.docx]

| Matrix for creating an overview of self-determination support strategies | | |
| --- | --- | --- |
| Strategy | Programs in Dutch care services *(*Grote methodiekendek 2022) | International literature |
| 1. Equal cooperation between relatives and healthcare professionals | LACCS  PERSPECTIEF  Active support | Kúld et al., 2023 review of intervention studies |
| 1. Sensitivity | LACCS  PERSPECTIEF  Pact  Verstandbaar maken  Active support | Kúld et al., 2023 review of intervention studies |
| 1. Purposeful application of total communication | LACCS  PERSPECTIEF  Pact  Verstandbaar maken  Active support | Kúld et al., 2023 review of intervention studies |
| 1. Choice making | LACCS  PERSPECTIEF  Pact  Verstandbaar maken  Active support | Kúld et al., 2023 review of intervention studies |
| 1. Implementation of technology |  | Kúld et al., 2023 review of intervention studies |
| 1. Creating and maintaining a network of acquaintances | LACCS  PERSPECTIEF | Kúld et al., 2023 review of intervention studies |

Description of object definitions/treatment elements from the above resources: This matrix was made on the basis if the de Distallation and matching method of Chopita and Leiden (2009):

The literature and practice element were extracted using the PracticeWise Clinical Coding System (PracticeWise, 2005), summarizing multiple variables pertaining to studies/interventions: 1) study groups (participants/problems), 2) treatment protocols (treatment). A practice element was defined as a discrete clinical technique or strategy (e.g., time out, relaxation) used as part of a larger intervention plan/begeleidingsmethode. Treatment operations were coded in line with availability of the descriptions of such variables in the protocol sources (aliade boek/vilans website) that matched with the results of previous research within the project, for example the code for ‘problem’ was matched with words or sentences describing self-determination. This resulted in the following codes:

1. Study groups: people with S/PIMD
2. Problem: target area: self-determination, own control, self-determination, having influence on their lives, making decisions, being understood by others, connecting others to the signals/behaviour and the needs of people with S/PIMD.
3. Studies: description of treatments supported by research
4. Practice element: description of what a support person(s) do to reach improved self-determination.

Programs in Dutch care services *(*Grote methodiekendek 2022)

**LACCS:**

Equal cooperation relatives/professionals:

With the client's developmental phase as a basis, the five LACCS areas (:1) Physical well-being; 2) Alertness; 3) Contact; 4) Communication; 5); Stimulating use of time) are examined during the good-life conversation, in which relatives, supervisors, therapists and the behavioral scientist involved participate. A questionnaire about the LACCS areas creates a complete picture of the client and his/her life and needs. All those involved around the client have a stimulating basic attitude and work together methodically.

Sensitivity

Based on the cooperation/conversations, a complete picture of the client, his/her needs and the meaning of behavior/signals is created.

Purposeful application of total communication/support and stimulation of making choices:

To support communication and making choices, three developmental phases must be distinguished: Sensation phase: Client is in a phase of sensory perception in the present moment. · Click phase: Client can understand a connection between events. If this… then that. · Understanding phase:

Client is in a phase in which he/she knows that things go in a certain way (routines), and also knows why that is the case. Insight and understanding are part of this phase. The client can consciously adjust his/her behavior to a situation and take others or the circumstances into account.

Creating/maintaining a network of acquaintances

The client's social network is actively involved in various regular conversations: Development phase conversation and a good-life conversation. Based on the results of these conversations, the image formation is determined again and again by what the client shows and goals and activities are adjusted to this.

**Perspectief:**

Equal cooperation of relatives and professionals:

Interdisciplinary, systematic and goal-oriented cooperation: professionals (residential and activity supervisors, behavioral scientists, other therapists and managers) and relatives work on the same goals from the same vision. Uses imaging, goals, activities, reports and evaluations.

Developing sensitivity:

Building a relationship, in which every behavior is a signal from the client seen as a deliberate attempt to say something. The program is based on the vision that people with ZEVMB can enter into a relationship with their environment and thus clarify their wishes and needs. The individual plan of the client is always adapted to his needs, wishes and possibilities.

Purposeful application of total communication

The goal is to help the person to learn new things and apply skills in different situations. Preventing deterioration is also development. Based on evaluations, the individual plan is adapted to the needs, wishes and possibilities of the client.

Supporting and stimulating making choices:

Based on complete picture, the individual plan of the client is made and always adapted to his needs, wishes and possibilities.

Creating/maintaining a network of acquaintances

Detailed information is collected about the client: image formation, goals, activities, reports and evaluations with certain questionnaires and instruments. Relatives and professionals keep in regular contact.

**Persoonlijk Activeringsprogramma (PAct) (Please note: PACT was developed within the same department as Perspectief, and has the same working method, but focuses on activities within the day centre).**

Equal cooperation of relatives and professionals:

Those involved work together systematically and purposefully to create an activity offer that matches the possibilities and preferences of the client. Unclear when relatives are involved.

Developing sensitivity: Building a relationship, collecting information about the possibilities/preferences of the client, and how these can best be implemented.

Purposeful application of total communication/Supporting and stimulating choice making: stimulating, connecting with the possibilities and preferences of the client.

**Verstaanbaar maken**

Developing sensitivity: Supervisors are sensitive and responsive and consider their interpretation of clients' behavior as a hypothesis that has been tested in practice. Looking at behavior/signals on a small scale. Systematic video analysis has been used to observe and interpret behavior and signaling.

Purposeful application of total communication/ Supporting and stimulating choice making: Supervisors are prepared to immerse themselves in clients and learn about their way of communicating in a course. Supervisors gain insight into the importance of their own behavior and forms of communication and to adjust these to the pace, possibilities of the client and by taking into account the amount and duration of the sensory stimuli offered and pitfalls (such as crowds in the environment).

**Active support**

Equal cooperation of relatives and professionals: The client's social network is actively involved in the image formation that consists of: talent poster, instruction plan, activity plan - this creates the image that can always be created in a cyclical process based on changes in the client.

When filling in the talent poster, the client, the social network and supervisors are involved if possible. Positive characteristics, skills (and preferences and interests) inventoried in cooperation with people in the client's environment.

Developing sensitivity: All behavior is communication and all communication is behavior.

Purposeful application of total communication: The goal of the instruction plan is for the client to learn and develop new skills step by step. It is focused on successful experiences and must therefore be described in achievable goals. Yak analysis: dividing a task or activity into small steps. The instruction plan is in line with the client's learning style and is aimed at increasing his/her capacities and self-direction. The individual possibilities and a stimulating environment are also taken into account. Activity plan: realizing a varied day with activities that are meaningful to the client, alternating effort and relaxation. Predictability is important here; All supervisors follow the activity plan and the activity plan is made clear to the client in a way that suits his/her communication capabilities.

Supporting and encouraging choice-making: In the positive image formation, the preferences and interests of the client are inventoried. Supervisors ask the client and the people in his/her environment for this. The opportunity plan ties in with the preferences and interests of the client, leaves room for his/her own choices and contributes to his/her empowerment.

| **Overview** | | | |
| --- | --- | --- | --- |
| **Review (Kúld et al., 2023)** | | **Concept mapping study (Kúld et al., 2023)** | **Delphie study (Nijs et al., 2022)** |
| **Self-determination component** | **Intervention element** | **Clusters** | **Statements from panellists.** |
| **Choice making** | | | |
| Choice making  *Food, drinks, leisure and occupational activities, hygiene, clothing, self-care, work activities, social interaction* | - Microswitches - Picture exchange system - Pairing objects to activities - Smart phones, iPads, applications - Optical pointers or sensors - Textured symbols - ACC systems - Multiliteracies training - Driving to learn an electric wheelchair | - Have a vision regarding usage of aids to aid choice making - Active stimulate choice making with communication - Implementing technology to stimulate   choice making | All respondents (strongly) agreed that some concepts (making choices, making decisions, being seen, experiencing the environment, problem solving) are important or vital for the self-determination.  Simple decisions (e.g., which toy to play with, what to eat, drink etc). |
| Summary: these elements overlap due to focus on choice making. The intervention elements (review) were used as an aid in choice making. The elements of the concept mapping study also focus on choice making through aids. The Delphie study statement emphasizes choice making as a vital part of self-determination. Strategy: choice making | | | |
| **Purposeful application of total communication** | | | |
| Communication | - Multiliteracties training (language of choice). - Microswitches - Picture exchange system - Pairing objects to activities - Smart phones, iPads, applications - Optical pointers or sensors - Textured symbols - ACC systems - Interactive robots | - Active communication in daily situations - Importance of clarity and predictability in daily communication and treatment programs - Actively support communication with aids and technology   Offer space and time for communication using aids that fit the individual | “there are other ways to communicate than verbal” (round 3). “If you know a person’s behavior repertoire and ways of communicating you could be able to see, you do not need verbal language” (round 2). Moreover, “a lot can be deduced by observing behavioral reactions of persons with (round 2) and “familiarity and sensitivity help in interpreting communication without words”  Using non-verbal communication by interpreting and knowing the persons behaviour repertoire and ways of communication. |
| Summary: These elements overlap as they focus on communication. In the review and concept mapping study it was emphasized that communication can be improved via aids, and via observation, interpretation of behaviour and other non-verbal ways (Concept mapping study and Delphie study). **Strategy**: purposeful application of total communication. | | | |
| **Implementation of technology** | | | |
| Technology and aids | - Microswitches - Picture exchange system - Pairing objects to activities - Smart phones, iPads, applications - Optical pointers or sensors - Textured symbols - ACC systems - Multiliteracies training - Driving to learn an electric wheelchair | Have a vision on the usage of technology  Implement technology  Stimulate choices and communication with technology |  |
| Summary: the focus of these element is to aid in choice making and communication via technology and aids. In the Delphi study of Nijs (2022), choices and communication were underlined without mentioning aids as this study focused on the operationalization self-determination. | | | |
| **Sensitivity of communication partner** | | | |
| Sensitivity to interpret behaviour and signals and respond to them | - Training caretakers to recognize and respond to client’s acceptance and rejection behaviours - Training caretakers to recognize choice making opportunities (moments where the person seems to be alert to make choices) and offer choices (and to read the person’s response). | - Give meaning to the signals of the person and reflect on these meaning. - Be sensitive by looking at the details in the persons behavior or reactions. - Observe, interpret and on the basis of this to and respond. | Sensitive and supportive persons in the environment are needed to provide the person with self-determination.  It is important that the persons in the environment are sensitive to the behaviour, skills, and preferences of the person with  Sensitive and supportive persons in the environment are needed to provide persons opportunities for self-determination; persons in the environment must be careful in interpreting the behaviour of persons with in order to not underestimate their abilities.  To understand the person with it is important that caregivers are familiar with the persons idiosyncratic responses. “Familiarity and sensitivity help in interpreting communication without words” |
| Summary: These elements overlap as they emphasize the importance of significant others (caretakers, relatives) to be sensitive. **Strategy:** develop sensitivity. | | | |
| **Strategy: building and maintaining a an network of acquaintances** | | | |
|  | - Training of relatives’ and caregivers’ supporter responsiveness - Preference assessment - Interpret and respond to behaviour of a person with disability to understand their preferences. - Get to know the person with disability. - Get a clear picture of the person with disability. - Keep records of the persons preferences. | - Sufficient and complete understanding of the person - Knowing the uniqe wishes and needs of my family member with . - See my family member   • Getting to know the person with (Z)EVMB and connecting with him/her  • Conditions for really getting to know the person with (Z)EVMB Collaboration between relatives and professionals who learn from each other | Familiarity is important in the relationship between the person with profound intellectual and multiple disabilities and the environment to support self-determination.  It is important that caregivers are familiar with the persons idiosyncratic responses. Familiarity and exchange of tacit knowledge can help persons in the environment to interpret the behaviour of persons with and provide them opportunities to live a self-determined life. Familiarity and sensitivity help in interpreting communication without words. |
| Summary: these elements overlap as they emphasize being familiar with the person e.g., knowing and understanding their abilities, capabilities, needs and preferences, and the meaning of their idiosyncratic behavior and responses to be able to react on these appropriately and provide them with an environment where self-determination is supported. | | | |
| **Strategy: collaboration of involved parties** | | | |
| Supported decision making  Individuality | - Training of relatives’ and caregivers’ supporter responsiveness, for them to work together to;   Interpret and respond to behaviour of a person with disability to understand their preferences.  Get to know the person with disability.  Get a clear picture of the person with disability.  Keep records of the persons preference.  Relatives and caregivers work together using the retrieved knowledge to make decisions on behalf of the person with disabilities.  Caregivers share the retrieved knowledge with other caregivers. | • Good and complete image of people with ZEVMB  • Together with all those involved, get to know someone from all contexts  • When relatives and care professionals work together on an optimal context to be able to make choices themselves  • Know the unique wishes and needs of my family member  • See my family member  • (Get to know) the person with (Z)EVMB and connect with him/her  • Conditions for really getting to know the person with (Z)EVMB  • Given the context, coordinate expectations between parents and care professionals  • In consultation, coordinate expectations between parents and care professionals about the person with (Z)EVMB | ..exchange of tacit knowledge can help persons in the environment to interpret the behaviour of persons with and provide them opportunities to live a self-determined life.  Many concepts of self-determination are only feasible if the caregiver or teacher provides the right environment. For persons with , it is unlikely to be self-determined, unless there is an interdependency between them and a facilitator, support system, and/or a tool.  It is the task of the persons in the environment to create an optimal situation for persons with . The persons in the environment must create opportunities so persons with have as much influence as possible and activities need to be scaffolded within the possibilities of the person with . |
| Engagement in meaningful relationships | - Person centred active support - Change in living situation - Joint painting procedure (autonomy within relational construct of mother and child with ). | - Building and maintaining a relationship   Experiencing the world through contact with others | …For many, options are only understood through experiencing them, or through the projection of partners. |
| Summary: These elements seem to overlap with the elements in the columns above due their focus on the role of the environment (significant others). However, they are put separate from the columns above because they focus overall on the role of significant others to **work together** towards; **getting to know the person with , to take account of the individuality of each person with**  (forming a complete picture, familiarity), **exchange knowledge** between significant others and with these information **build and provide an environment** where there are opportunities for self-determination. These elements overlap because of their focus on relationships. The intervention elements from the review focused on improving relationships of people with to significant others. The elements from the concept mapping study focus on forming and sustaining relationships and that people with experience the world through contact with others, which fits with the one statement from the Delphie study stating that options are understood through projection of partners. | | | |

De Grote Methodiekengids. (2024). University of Groningen Press

Kúld, P. B., Frielink, N., Schuengel, C., & Embregts, P. J. C. M. (2024). Supporting self-determination of individuals with severe or profound intellectual and multiple disabilities according to relatives and healthcare professionals: A concept mapping study. *Journal of applied research in intellectual disabilities : JARID*, *37*(4), e13267. https://doi.org/10.1111/jar.13267

Kúld, P. B., Frielink, N., Zijlmans, M., Schuengel, C., & Embregts, P. J. C. M. (2023). Promoting self-determination of persons with severe or profound intellectual disabilities: a systematic review and meta-analysis. *Journal of Intellectual Disability Research : Jidr, 67*(7), 589–629. https://doi.org/10.1111/jir.13036

Nijs, S., Zijlmans, M., Schuengel, C., & Embregts, P. J. C. M. (2022). Operationalisation of self-determination of persons with profound intellectual and multiple disabilities: a Delphi study. *Journal of Intellectual & Developmental Disability*, 1-13, 1–13. <https://doi.org/10.3109/13668250.2022.2147053>

Vilans erkende methodes (n.d.). *Databank interventies*. Retrieved June 30, 2025, from <https://www.databankinterventies.nl/>
